# Acute effects of a single unilateral balance training session on ipsi- and contralateral balance performance in healthy young adults

**DOI:** 10.1186/s13104-021-05774-7

**Published:** 2021-09-10

**Authors:** Thomas Muehlbauer, Leander Abel, Simon Schedler, Stefan Panzer

**Affiliations:** 1 Division of Movement and Training Sciences/Biomechanics of Sport, University of Duisburg-Essen, Gladbecker Str. 182, 45141 Essen, Germany; 2 Institute of Sport Science, Saarland University, 66123 Saarbrücken, Germany; 3 Department of Health and Kinesiology, Texas A&M University, College Station, TX USA

**Keywords:** Postural control, Inter-limb transfer, Side-to-side difference, Leg asymmetry

## Abstract

**Objective:**

While there is evidence on the short-term effects of unilateral balance training (BT) on bipedal balance performance, less is known on the acute effects of unilateral BT on unilateral (i.e., ipsi- and contralateral) balance performance. Thus, the present study examined the acute effects of a single unilateral BT session conducted with the non-dominant, left leg or the dominant, right leg on ipsilateral (i.e. retention) and contralateral (i.e., inter-limb transfer) balance performance in healthy young adults (*N* = 28).

**Results:**

Irrespective of practice condition, significant improvements (*p* < 0.001, *d* = 1.27) in balance performance following a single session of unilateral BT were observed for both legs. Further, significant performance differences at the pretest (*p* = 0.002, *d* = 0.44) to the detriment of the non-dominant, left leg diminished immediately and 30 min after the single unilateral BT session but occurred again 24 h following training (*p* = 0.030, *d* = 0.36). These findings indicate that a single session of unilateral BT is effective to reduced side-to-side differences in balance performance, but this impact is only temporary.

## Introduction

Previous studies [1, 2] have shown that unilateral balance training (BT) is effective to induce balance enhancements in the trained as well as the untrained limb. A closer look at these and further studies however, shows that the effects were mainly achieved through the long-term use (i.e., several weeks or months) of unilateral BT. In contrast, there are comparatively few studies [3, 4] that examined the short-term application (i.e., single session) of unilateral BT (i.e., acute effects). However, in these studies during the pre- and posttest bipedal but not unipedal balance performance was assessed and varying findings were reported. More precisely, Hammami et al. [3] tested adolescent volleyball players (mean age: 14 years) before and after three different unilateral balance protocols (i.e., anterior, mediolateral, and rotational balance exercises) each lasting 15 min. They found better bipedal balance performance (i.e., decreased area of postural sway) after the anterior but worse postural control (i.e., increased area of postural sway) following the mediolateral and the rotational exercise mode. Further, Romero-Franco et al. [4] detected significant decrements in bipedal balance performance (i.e., increased length and speed of postural sway) following a 25-min. unilaterally performed proprioceptive exercise session in adult athletes (age range: 17–33 years). The two aforementioned studies have expanded the knowledge on the acute effects of unilateral BT on bipedal balance performance. However, the question arises to what extent a short-term unilateral BT is effective to induce balance enhancements in the trained, ipsilateral (i.e., retention) and the untrained, contralateral (i.e., transfer) limb, separately. Furthermore, the question arises whether a single unilateral BT session with the dominant versus the non-dominant limb produces different effects. Preliminary evidence for the dependency of retention (i.e., trained, ipsilateral limb) and inter-limb transfer (i.e., untrained, contralateral limb) effects with regard to the leg used for unilateral BT was provided in a recently published study. Marcori et al. [5] compared balance performance in adults (age range: 18–30 years) following a single unilateral BT session (10 min. in total) that was performed with the non-dominant, left or the dominant, right leg. Immediately after BT, both groups achieved similar balance improvements with the trained leg and equivalent performance inter-limb transfer to the untrained leg. However, in the 24 h delayed posttest the group that trained with the dominant, right leg showed superior inter-limb transfer to the untrained leg than vice versa.

The aim of this study was to replicate the aforementioned findings by examining the acute effects of a single unilateral BT session performed with the non-dominant, left or the dominant, right leg on ipsi- (i.e., retention) and contralateral (i.e., inter-limb transfer) balance performance in healthy young adults. Based on the concept of hemispheric lateralization stating asymmetric transfer across limbs [6] and with reference to the relevant literature showing that right hemisphere is responsible for quiet and perturbed postural control [7], we expected that both exercise conditions will result in enhanced balance performance but the inter-limb transfer of the training effects will be larger for the dominant, right leg BT group compared to the non-dominant, left leg BT group. The present topic is important from both a theoretical and practical perspective. On the one hand, further insights into processes of postural control underlying side-dependent short-term balance practice will be obtained and on the other hand, practitioners will be provided with information about the most effective practice order for inter-limb transfer.

## Main text

### Methods

#### Participants

Twenty-eight young adults (sex: 14 men, 14 women; age: 23 ± 4 years; stature: 174.5 ± 8.5 cm; weight: 69.7 ± 10.0 kg) participated in this study and were randomly assigned to one of two groups (*n* = 14 each) with similar numbers of men and women, i.e., non-dominant, left leg BT group or dominant, right leg BT group. All participants self-declared their right leg as dominant leg used for kicking a ball. None of the participants reported a history of musculoskeletal or neurological disorder or injury. The study protocol was approved by The Human Ethics Committee at the University of Duisburg-Essen, Germany.

#### Study design and procedure

**Fig. 1:**
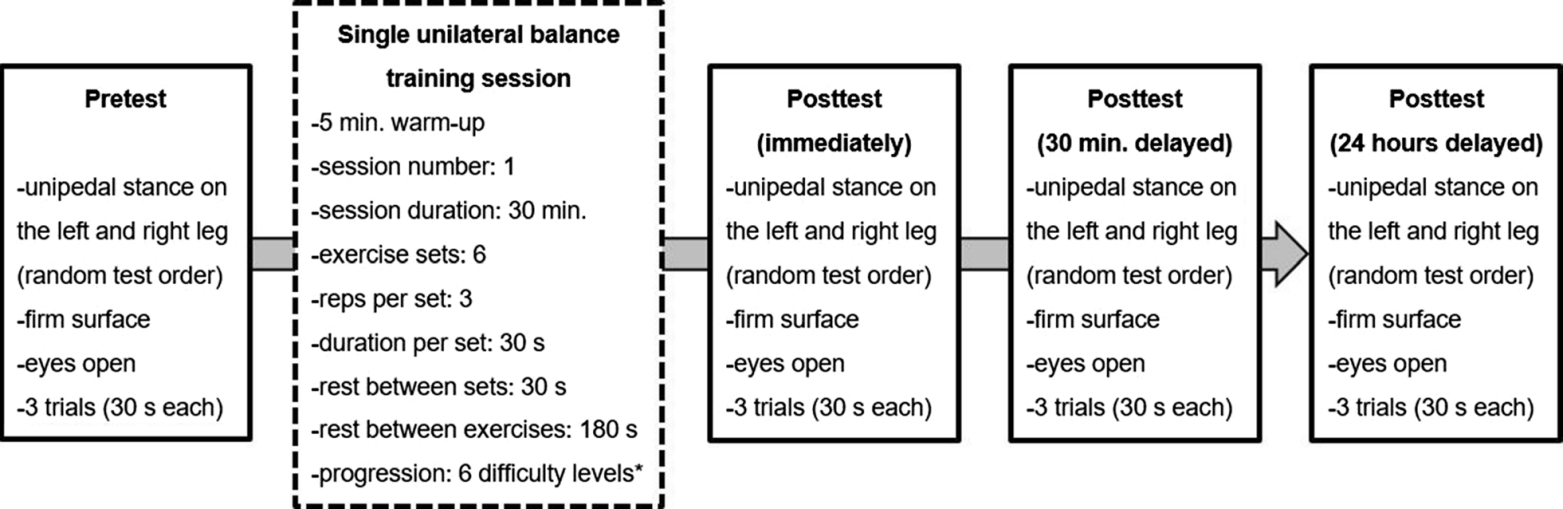
Schematic description of the study design. *A balance board (Wobblesmart^©^, Artzt GmbH, Dornburg, Germany) was used that is equipped with a mechanically adjustable pivot to increase task difficulty from level 1 (low) to 6 (high) by reducing the base of support diameter

The study was conducted across five phases consisting of a pretest, a single unilateral BT session, an immediate posttest, a 30 min. delayed posttest, and a 24 h delayed posttest (Fig. 1). Upon entering the laboratory, all participants received standardised verbal instructions and visual demonstrations regarding the test and training procedure.

#### Assessment of balance performance

Balance performance was assessed while performing the unipedal stance before (pretest) and three times (immediate posttest, 30 min. delayed posttest, 24 h delayed posttest) after the unilateral BT session (Fig. 1). More specifically, participants had to stand on one leg without shoes while the other leg was flexed to ~90°. Further, they had to place their hands on the hips and to fixate a cross marked at eye level on a nearby wall. Participants were instructed to stand with eyes open for maximal 30 s. Three test trials per leg were conducted and the best trial (i.e., least postural sway) was used for further data analysis. The sequence of the tested leg was randomly alternated across participants. A force plate (AMTI AccuSway, Watertown, USA) measuring the ground reaction forces was used to determine the centre of pressure (CoP) displacement length during each trial. Data was sampled at 100 Hz and processed using a fourth-order Butterworth low-pass filter with a cut-off frequency of 10 Hz.

#### Unilateral balance training session

The unilateral BT consisted of a single 30 min. session with one group that used their non-dominant, left leg and the other group that used their dominant, right leg (Fig. 1). The rationale for using only one training session was that previous research showed structural and functional brain changes after relatively short periods of BT (i.e., after 2 sessions using 15 × 30 s trials each) in healthy young adults [8]. Moreover, a single BT session is performed by athletes, for example, as part of their warm-up to improve performance or to prevent injury before a competition. After a 5 min. warm-up including running at light intensity, all participants performed the unipedal stance using a balance board. The board is equipped with a height-adjustable pivot to increase task difficulty from level 1 (low) to 6 (high) by a continuous reduction of the base of support diameter from ~14 to 4 cm [9]. Three repetitions (30 s per rep) per difficulty level/set were conducted with a 30 s rest period between sets and a 180 s break between exercises.

#### Statistical analysis

The data was analysed (SPSS 27.0, IBM Statistics, USA) by a 2 (group: non-dominant, left leg practice; dominant, right leg practice) × 2 (leg: non-dominant, left leg; dominant, right leg) × 4 (test: pretest; immediate posttest; 30 min. delayed posttest; 24 h delayed posttest) ANOVA with repeated measures on leg and test. If a significant interaction effect occurred, post-hoc tests were computed. The significance level was set at *p* < 0.05. The effect size measure Cohen’s *d* was calculated and classified as small (0 ≤ *d* ≤ 0.49), medium (0.50 ≤ *d* ≤ 0.79), and large (*d* ≥ 0.80) [10].

### Results

**Fig. 2:**
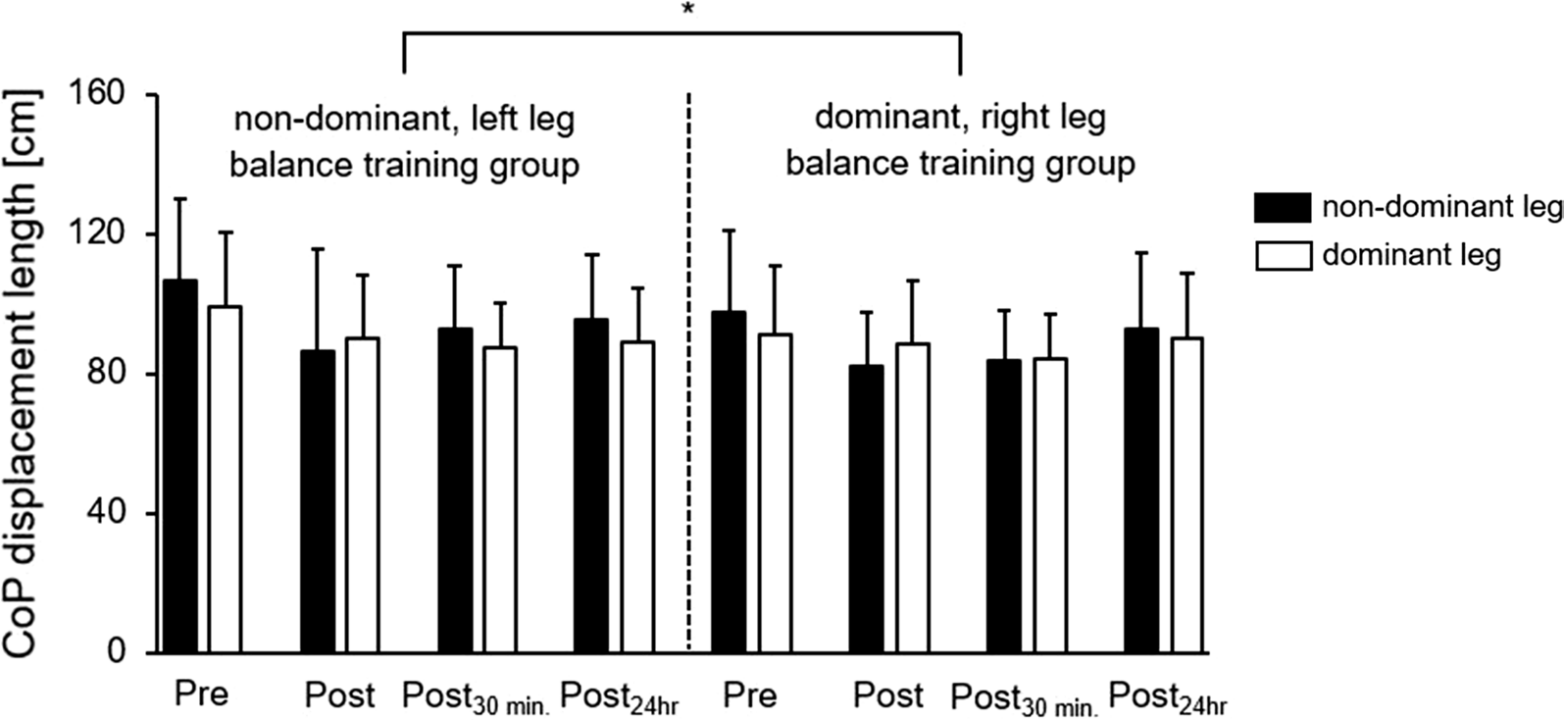
Means and standard deviations of the CoP displacement length for the non-dominant, left leg and the dominant, right leg by training group across the experimental phases: pretest, immediate posttest, 30 min. delayed posttest, 24 h delayed posttest. *Significant Leg × Test interaction (*p* = 0.007, *d* = 0.82) in favour of the dominant, right leg at pretest and 24 h delayed posttest that was irrespective of group

Balance performance for the non-dominant, left leg and the dominant, right leg by training group across the experimental phases are illustrated in Fig. 2. The descriptive results showed performance improvements from the pretest to the subsequent posttests of + 10 to + 19% (non-dominant leg) and + 9 to + 12% (dominant leg) for the non-dominant, left leg practice group and of + 5 to + 16% (non-dominant leg) and + 1 to + 8% (dominant leg) for the dominant, right leg practice group. Compared with previous acute BT studies, similar enhancements (left leg practice group: + 23.7 to + 31.5% for the non-dominant leg and + 12.8 to + 20.1% for the dominant leg; right leg practice group: + 9.2 to + 23.1% for the non-dominant leg and + 24.5 to + 30% for the dominant leg) were reported by Marcori et al. [5] but contrary to that mainly decrements of − 5 to − 15% and − 32 to + 9% were stated by Romero-Franco et al. [4] and Hammami et al. [3], respectively. The additional comparison with chronic BT studies again shows similar improvements of + 5 to + 35% reported by Oliveira et al. [1] and + 8 to + 11% stated by Schlenstedt et al. [2]. The ANOVA yielded significant main effects of Test (*F*_(3, 78)_ = 10.462, *p* < 0.001, *d* = 1.27) and Leg (*F*_(1, 26)_ = 4.386, *p* = 0.046, *d* = 0.82) but not of Group (*F*_(1, 26)_ = 0.509, *p* = 0.482, *d* = 0.28). Further, a significant Leg × Test interaction (*F*_(3, 78)_ = 4.327, *p* = 0.007, *d* = 0.82) was detected. The post-hoc analysis revealed significantly better balance performance for the dominant, right leg compared to the non-dominant, left leg during the pretest (*p* = 0.002, *d* = 0.44) and the 24 h delayed posttest (*p* = 0.030, *d* = 0.36). Lastly, the Group × Leg interaction (*F*_(1, 26)_ = 2.349, *p* = 0.137, *d* = 0.60), the Group × Test interaction (*F*_(3, 78)_ = 1.017, *p* = 0.390, *d* = 0.40), and the Group × Leg × Test interaction (*F*_(3, 78)_ = 0.183, *p* = 0.907, *d* = 0.17) did not reach the level of significance.

### Discussion

In accordance with the first part of our hypothesis stating that both practice conditions will result in enhanced postural control, the significant main effect of Test indicated that the two groups improved their balance performance (i.e., reduced CoP displacements) in both legs. This finding corresponds with those from previous studies examining the acute [5] or chronic [1, 2] effects of unilateral BT on unipedal balance performance. In contrast to the second part of our hypothesis assuming that the training effects will be larger for the dominant, right leg BT group compared to the non-dominant, left leg BT group, no significant Group × Leg × Test interaction was detected. This indicates that the training-related performance changes after a single unilateral BT session did not depend on the limb used for practice. Our finding is contrary to the results of Marcori et al. [5] who detected a significant Group × Leg × Test interaction stating a greater performance transfer from the dominant, right to the non-dominant, left leg. What is a likely reason for the discrepancy between our findings and those of Marcori and colleagues? At the pretest, Marcori et al. [5] reported no performance discrepancies between the legs whereas a significant difference to the detriment of the non-dominant, left leg was detected in the present study. This imbalance disappeared immediately as well as 30 min after the BT session, but was again observed 24 h after training. On the one hand, this pattern of results shows that a single unilateral BT session is effective to reduce side differences at least temporarily. On the other hand, the significantly poorer baseline level in the non-dominant, left leg compared to the dominant, right leg is indicative for a larger adaptive reserve of the former one. This in turn could have an effect on the inter-limb transfer between the non-dominant and the dominant leg. In fact, Teixeira et al. [11] showed that soccer practice performed with emphasis on the non-dominant leg led to significant improvements in the non-dominant and the dominant leg. In contrast, practice with emphasis on the dominant leg resulted in significant enhancements in the dominant leg only. Therefore, future studies should compare the acute effects of unilateral BT in individuals with a dominant, right leg versus those with a dominant, left leg. This would help to clarify whether there is actually a better inter-limb transfer from the right to the left side or rather from the non-dominant to the dominant side.

### Conclusion

The present study investigated the acute effects of a single unilateral BT session conducted with the non-dominant, left leg or the dominant, right leg on ipsilateral (i.e., retention) and contralateral (i.e., inter-limb transfer) balance performance in healthy young adults. Both practice conditions resulted in significantly improved balance performance in the two legs, whereby the observed performance changes following training did not depend on the limb used for practice. The additionally observed disappearance of performance differences between the legs (i.e., worse for the non-dominant, left leg at the pretest) immediately and 30 min but not 24 h after training refers to a temporary reduction of side-to-side differences as a result of a single unilateral BT session.

### Limitations

There are a few limitations of this study that need to be addressed. First, acute effects of a single unilateral BT session conducted with the non-dominant, left leg or the dominant, right leg was investigated, which limits the transferability of the present results to the long-term application of unilateral BT on ipsi- and contralateral balance performance. Thus, future studies should extend our approach and investigate the inter-limb transfer following several weeks or months of unilateral BT. Second, we examined balance performance on a behavioural level using CoP displacement, but we did not investigate the underlying neuromuscular mechanisms. Therefore, future studies are advised to include assessments of neuronal (e.g., changes in brain activation) and muscular (i.e., changes in muscle activity) correlates. Third, our analyses are limited to the CoP length, and thus further studies should examine what results will obtained when alternative outcome measures (e.g., CoP velocity) are used. Fourth, two intervention groups but no passive (no training) or active (alternative training) control condition was applied and should be considered in future studies.

## Data Availability

The data generated and analysed during the present study are available from the corresponding author upon reasonable request.

## Abbreviations

ANOVA: Analysis of variance
BT: Balance training
CoP: Centre of pressure
